# Quantification of the Impact of Feature Selection on the Variance of Cross-Validation Error Estimation

**DOI:** 10.1155/2007/16354

**Published:** 2007-02-19

**Authors:** Yufei Xiao, Jianping Hua, Edward R Dougherty

**Affiliations:** 1 Department of Electrical and Computer Engineering, Texas A&M University, College Station, TX 77843, USA; 2 Computational Biology Division, Translational Genomics Research Institute, Phoenix, AZ 85004, USA

## Abstract

Given the relatively small number of microarrays typically used in gene-expression-based classification, all of the data must be used to train a classifier and therefore the same training data is used for error estimation. The key issue regarding the quality of an error estimator in the context of small samples is its accuracy, and this is most directly analyzed via the deviation distribution of the estimator, this being the distribution of the difference between the estimated and true errors. Past studies indicate that given a prior set of features, cross-validation does not perform as well in this regard as some other training-data-based error estimators. The purpose of this study is to quantify the degree to which feature selection increases the variation of the deviation distribution in addition to the variation in the absence of feature selection. To this end, we propose the coefficient of relative increase in deviation dispersion (CRIDD), which gives the relative increase in the deviation-distribution variance using feature selection as opposed to using an optimal feature set without feature selection. The contribution of feature selection to the variance of the deviation distribution can be significant, contributing to over half of the variance in many of the cases studied. We consider linear-discriminant analysis, 3-nearest-neighbor, and linear support vector machines for classification; sequential forward selection, sequential forward floating selection, and the -test for feature selection; and -fold and leave-one-out cross-validation for error estimation. We apply these to three feature-label models and patient data from a breast cancer study. In sum, the cross-validation deviation distribution is significantly flatter when there is feature selection, compared with the case when cross-validation is performed on a given feature set. This is reflected by the observed positive values of the CRIDD, which is defined to quantify the contribution of feature selection towards the deviation variance.

## 

[1,2,3,4,5,6,7,8,9,10,11,12,13]
